# Curative effect and technical key points of laparoscopic surgery for choledochal cysts in children

**DOI:** 10.3389/fsurg.2022.1051142

**Published:** 2023-01-06

**Authors:** Zedong Bian, Yong Zhi, Xinhao Zeng, Xiaoyong Wang

**Affiliations:** ^1^ Department of Pediatric Surgery, Affiliated Hospital of Southwest Medical University, Luzhou, China; ^2^ Sichuan Clinical Research Center for Birth Defects, Luzhou, China

**Keywords:** choledochal cyst, laparoscopy, surgical procedures, hepaticojejunostomy, children

## Abstract

**Objective:**

The purpose of this study was to investigate the curative effect of and experience with laparoscopic surgery for congenital choledochal cysts in children.

**Methods:**

This is a retrospective analysis of 33 children diagnosed with congenital choledochal cyst in the pediatric surgery department of the Affiliated Hospital of Southwest Medical University between January 2019 and December 2021. The cohort included 8 males and 25 females aged 0.25–13.7 years (median age, 3.2 years), including 21 cases of type I and 12 cases of type IV choledochal cyst (Todani classification). Laparoscopic choledochal cyst resection and hepaticojejunostomy with Roux-en-Y anastomosis were performed in all the patients.

**Results:**

Laparoscopy without transit opening was successfully performed in the 33 cases. The duration of the procedure was 235–460 min (mean ± SD, 316 ± 61 min), and intraoperative blood loss volume was 15–40 ml (23 ± 7.6 ml). Postoperative hospital stay was 7–14 days (9 ± 1.8 days). Postoperative biliary fistula and pancreatitis occurred in two cases each, and all four patients were successfully treated with conservative treatment. No anastomotic stenosis, delayed bleeding, cholangitis, intestinal obstruction, or other complications occurred. All the children were followed up for 2–36 months (median period, 17.2 months). The clinical symptoms disappeared, and no obvious hepatic dysfunction was found on abdominal color ultrasound and liver function examination.

**Conclusion:**

Laparoscopic surgery for congenital choledochal cyst in children is safe and effective, as it is a minimally invasive surgery that is associated with a low degree of trauma and bleeding, rapid postoperative recovery, and satisfactory aesthetic appearance.

## Introduction

Choledochal cyst, also known as congenital biliary dilatation, is a common congenital biliary malformation in children (1, 2). The incidence rate in Asian countries is much higher than that in Europe and the United States: the incidence rate is about 1/1,000 in Japan, about 1/13,000 in other parts of Asia, and 1/100,000–150,000 in Europe and the United States (3, 4). In recent years, the incidence of choledochal cyst has been on the rise (5). The common clinical signs are interstitial upper abdominal pain, upper abdominal mass, jaundice, nausea, vomiting, and anorexia (6). If it is not treated on time, it could lead to pancreatitis, suppurative cholangitis, cyst rupture and perforation, repeated biliary tract infection, biliary tract obstruction, cirrhosis, and other serious complications (7).

Radical surgery is the only reliable and effective treatment, and the standard approach is choledochal cyst resection and hepaticojejunostomy with Roux-en-Y anastomosis (8, 9). However, traditional open surgery is associated with a high degree of trauma and bleeding, slow recovery, scarring, and a high incidence of postoperative intestinal adhesions and other complications (10). In 1995, Farello et al. first reported laparoscopic total choledochal cyst resection (11). Compared with open surgery, laparoscopic surgery is associated with a lower degree of trauma, shorter hospital stay, faster postoperative recovery, and better exposure of the surgical field (12–14). Further, as it is easy to observe the relationship between the cyst and surrounding tissue, the procedure can be performed with better accuracy and is safer (12–14). As a result of the continuous improvements in minimally invasive surgical instruments and surgical techniques, laparoscopic total choledochal cyst resection is now more widely applied in children. However, successful completion of laparoscopic total choledochal cyst resection requires a high level of skill in laparoscopic techniques and in-depth technical knowledge of the procedure. In order to contribute to the knowledge about this technique in the literature, the present study attempts to analyze and summarize the clinical data for 33 cases of laparoscopic choledochal cyst resection and hepaticojejunostomy Roux-en-Y anastomosis performed at the Pediatric Surgery Department of the Affiliated Hospital of Southwest Medical University between January 2019 and December 2021. Further, the surgical experience and techniques are discussed.

## Methods

### Patients

This retrospective analysis included the data for 33 children who underwent laparoscopic choledochal cyst resection and hepaticojejunostomy with Roux-en-Y anastomosis at the Pediatric Surgery Department of the Affiliated Hospital of Southwest Medical University between January 2019 and December 2021. The cohort included 8 males and 25 females with a median age of 3.2 years (0.25–13.7 years), and 21 had type I and 12 had type IV choledochal cyst (Todani classification). All the children had a clear diagnosis of choledochal cyst based on preoperative ultrasound B examination and magnetic resonance cholangiopancreatography. There were no contraindications for laparoscopic surgery in any of the cases, and all the procedures were performed by the same surgeon.

The inclusion criteria were (1) a confirmed preoperative diagnosis of choledochal cyst and (2) successful completion of choledochal cyst resection and hepaticojejunostomy with Roux-En-Y anastomosis under laparoscopic guidance. The exclusion criteria were (1) stage I open surgery for choledochal cyst resection and (2) stage I external drainage of the choledochal cyst and stage II choledochal cyst resection.

### Technical information

**Figure 1:**
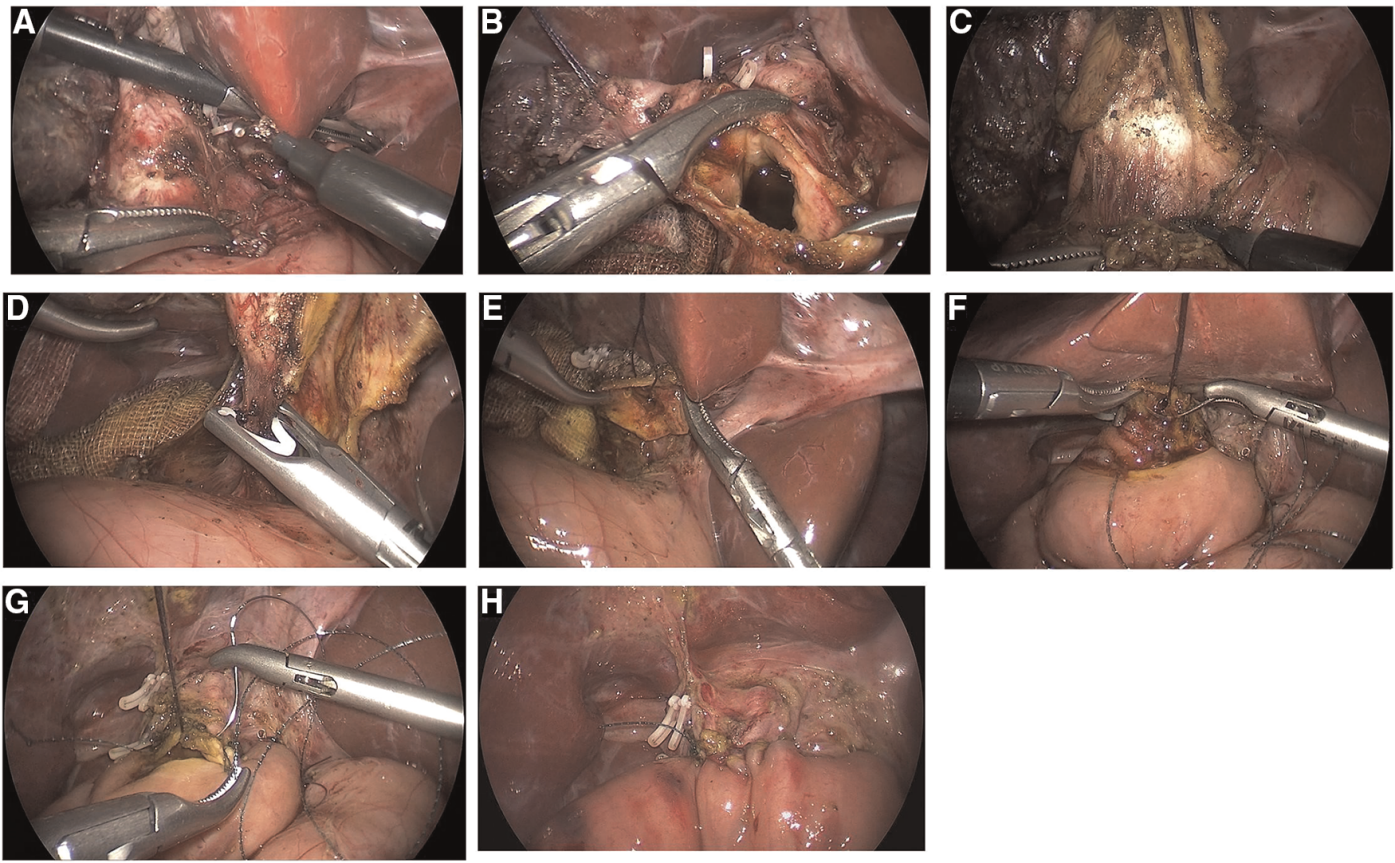
Images depicting the steps in total choledochal cyst resection and hepaticojejunostomy with Roux-en-Y anastomosis under laparoscopic guidance; the choledochal cyst was dissociated and exposed. (**A**) The choledochal cyst was dissociated and exposed. (**B**) Transverse incision of the anterior wall of the cyst was performed below the cystic duct opening, and the area was examined for vagal bile duct openings. (**C**) The cyst wall was gradually dissociated from the proximal end to the distal end. (**D**) The distal cyst wall was dissociated up to the thinner part close to the pancreatic duct, which was clamped and severed with the Hem-o-lock clip. (**E**) The opening of the common hepatic duct was trimmed. (**F**) The posterior wall of the common hepatic duct and the jejunum were sutured continuously from left to right (3–9 o’clock). (**G**) The anterior wall of the common hepatic duct and the jejunum were sutured continuously from left to right (3–9 o’clock). (**H**) Hepaticojejunostomy anastomosis was completed.

General anesthesia was induced with tracheal intubation. The patient was placed in a left-leaning supine position with the head raised. Pneumoperitoneum was established using the routine procedure, with the pressure set to 6–12 mmHg (1 mmHg = 0.133 kPa). A 10-mm trocar was used to create an opening for the laparoscope at the upper edge of the umbilicus. A 3- or 5-mm trocar was placed at three points: below the intersection of the left midline of the clavicle and costal margin, at the flat umbilicus on the outer edge of the right abdominus rectus muscle, and below the intersection of the right axillary front and costal margin. The ligamentum teres hepatis and the bottom of the gallbladder were suspended with a 3–0 absorbable suture, and pulled upward to fully expose the hepatic porta. An ultrasonic knife or electric coagulation hook was used to gradually dissociate the gallbladder from the base to the neck, ligate the gallbladder artery, and expose the choledochal cyst, as shown in Figure 1A. A transverse incision was made in the anterior wall of the cyst below the cystic duct opening, and decompression was performed to detect any vagal bile duct openings, as shown in Figure 1B. Following this, the posterior wall of the cyst was detached. The distal end of the cyst wall was lifted upward with a 3–0 absorbable suture, and the cyst wall was gradually dissociated from the proximal end to the distal end, as shown in Figure 1C, up to the thinner section close to the pancreatic duct, which was clamped and severed with the Hem-o-lock clip, as shown in Figure 1D. Next, the posterior wall of the proximal cyst was dissociated upward to the common hepatic duct, the remaining cystic wall was resected together with the gallbladder, and the opening of the common hepatic duct was trimmed, as shown in Figure 1E. At 10–15 cm from Treitz's ligament, the jejunum was lifted with a pair of intestinal forceps, dragged out of the abdominal cavity through the umbilical incision, and separated. An appropriate length of jejunal loop was determined according to the straight-line distance between the umbilical nest and hepatic porta (15–25 cm). The proximal jejunal loop was then closed, and the distal jejunal loop was anastomosed with the proximal jejunum. The jejunum was inserted into the abdominal cavity, and the pneumoperitoneum was re-established. Next, a tunnel was established in the vasculature-free area of the mesangium on the right side of the transverse colon, through which the jejunal loop was raised to the hepatic porta. The intestinal wall was incised at 1 cm from the blind end of the jejunal loop, and the length was consistent with the size of the opening of the common hepatic duct. A 4–0 barbed suture was used to suture the posterior wall of the common hepatic duct and jejunum continuously from 3 to 9 o’clock, as shown in Figure 1F. In the same way, a second barbed suture was used to suture the anterior wall continuously from 3 to 9 o’clock, as shown in Figure 1G, to complete hepaticojejunostomy anastomosis, as shown in Figure 1H. The abdominal cavity was rinsed, and a drainage tube was placed behind the anastomosis and extracted through an incision under the right costal margin. The trocars were removed, and the incision was sutured.

### Statistical analysis

The SPSS 22.0 software was used for statistical analysis. Measurement data were expressed as mean ± standard deviation (mean ± SD), and counting data were expressed as percentages.

## Results

Choledochal cyst resection and hepaticojejunostomy with Roux-en-Y anastomosis was performed under laparoscopic guidance without conversion in all the 33 cases of the present cohort. The operative time was 235–460 min (mean ± SD, 316 ± 61 min), and intraoperative blood loss volume was 15–40 ml (mean ± SD, 23 ± 7.6 ml). The postoperative hospital stay was 7–14 days (mean ± SD, 9 ± 1.8 days). Postoperative complications occurred in four cases (12.2%), including two cases of biliary fistula, and both were Grade I complications (Clavien-Dindo classification), the age was 2.5 and 5.7 year, and both were type I choledochal cyst (Todani classification), which was successfully treated with continuous unobstructed drainage without surgical treatment, and two cases of pancreatitis, and both were Grade II complications, the age was 3.5 (type IV) and 7.1 (type I) years, which was symptomatically treated by fasting, acid inhibition, pancreatic enzyme secretion inhibition, and parenteral nutrition support. None of the above 4 children had any definite adverse events during the operation. Anastomotic stenosis, delayed bleeding, cholangitis, intestinal obstruction, and other complications did not occur. All the children were followed up for 2–36 months (median period, 17.2 months) after the procedure. Over the follow-up period, the clinical symptoms disappeared and no obvious abnormalities were found on abdominal color ultrasound or liver function examinations, as shown in Table 1.

**Table 1:** Relevant data for the cohort (*N* = 33).

Item	
Number of cases (male/female)	33 (8/25)
Age (years), median (range)	3.2 (0.25–13.7)
Todani classification	
Type I, *n* (%)	21 (63.6)
Type IV, *n* (%)	12 (36.4)
Operation time (min), mean ± SD (range)	316 ± 61 (235–460)
Intraoperative blood loss (ml), mean ± SD (range)	23 ± 7.6 (15–40)
Postoperative complications	
Biliary fistula, *n* (%)	2 (6.1)
Pancreatitis, *n* (%)	2 (6.1)
Postoperative hospital stay (days), mean ± SD (range)	9 ± 1.8 (7–14)
Postoperative follow-up	
Follow-up time (months)	2–36
Hepatic dysfunction, *n* (%)	0 (0)
Imaging abnormality, *n* (%)	0 (0)

## Discussion

In the present cohort, choledochal cyst resection and hepaticojejunostomy with Roux-en-Y anastomosis under laparoscopic guidance was successfully performed in all 33 children, and the curative effect was satisfactory. This article describes the technical details and experiences during the procedure in order to the contribute to the knowledge available regarding this fairly difficult surgery. We hope that the information provided here will be useful to surgeons who perform this technique in the future.

**Figure 2:**
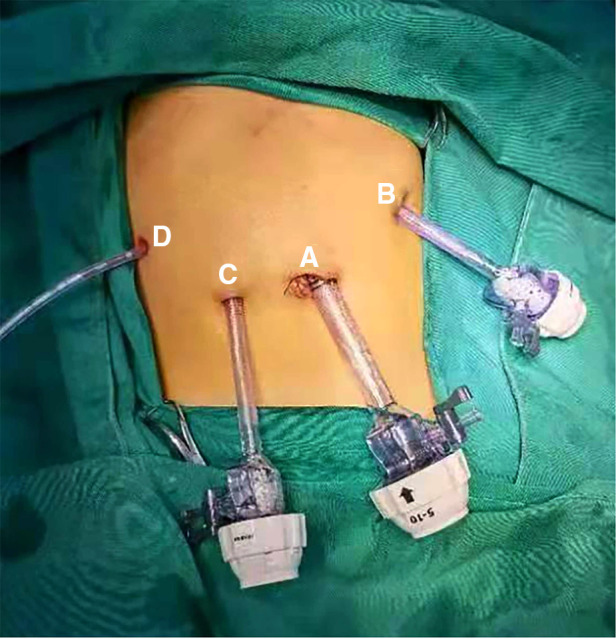
Intraoperative trocar and postoperative drainage tube layout. (**A**) Laparoscope hole (**B**) main operation hole (**C**) backup operation hole (**D**) auxiliary operation hole and postoperative drainage tube hole.

The main steps in laparoscopic choledochal cyst resection and hepaticojejunostomy with Roux-en-Y anastomosis are the stripping and removal of the choledochal cyst and reconstruction of the digestive tract. These steps are difficult to perform, and require an adequate trocar layout and good visual field exposure. For traditional porous laparoscopic total resection of the choledochal cyst, three to five trocars are inserted at the umbilicus and bilateral upper abdomen respectively (15–17). We modify this layout so that the puncture hole below the costal margin of the left midline of clavicle is used as the main operation hole for choledochal cyst resection and hepaticojejunostomy anastomosis, and the flat umbilical region at the outer margin of the right rectus abdominis is used as the backup operation hole. The surgeon stands on the left of the child, so that the angle between the two operating arms can be increased to a certain extent. This position is more convenient for the surgeon and reduces upper limb fatigue during the long procedure. In addition, the puncture hole under the costal margin of the left midline of the clavicle is closer to the plane of the choledochal cyst and is almost vertical to the longitudinal axis of the cyst. This improves precision and is convenient when the cyst is stripped from the top to the bottom through the hole. The puncture hole under the costal margin of the right axillary front is generally used as an auxiliary operation hole and is also the point at which the drainage tube is extracted, as shown in Figure 2. Due to the small abdominal cavity space in children, which is more obvious in infants, and the hindrance posed by the liver, the whole surgical field is not well exposed and it is difficult to perform the procedure under laparoscopic guidance. Therefore, good suspension and traction technology is required to achieve satisfactory surgical field exposure in laparoscopic total choledochal cyst resection in children. For this purpose, two 3–0 absorbable sutures with needles are used to create a V-shaped suspension at the gallbladder fossa and ligamentum teres hepatis, and the assistant lifts the traction wire outside the abdominal cavity to fully expose the structure of the porta.

Before the cyst is stripped, it is routine procedure to dissociate the gallbladder from the cystic fossa, ligate the cystic artery, and then dissociate the cystic duct until the confluence of the cystic duct and the cyst is completely exposed. At this time, the cystic duct is not temporarily separated as an anatomical marker. For patients with large cysts, it is not advisable to open the anterior wall of the cyst, in order to avoid accidental injury to the left and right hepatic duct. The anterior wall of the cyst should be opened under the cystic duct opening after the positions of the left and right hepatic duct opening are confirmed. Additionally, the cystic duct or cyst wall should be carefully checked for vagal bile duct openings, so as to prevent postoperative biliary fistula that could be caused by the omission of such openings during anastomosis. Additionally, intracapsular decompression may help to expose and suspend the cyst. Conventional suspension and traction should be performed when the anterior and posterior walls of the distal cyst are dissociated. For large cysts, traction at different levels of cyst wall is feasible, so the distal cyst can be gradually dissociated and then lifted up. This is conducive to exposing the gap between the cyst wall and pancreas or portal vein. Further, the cyst should be dissociated close to the cyst wall that is slightly above the junction of the cyst wall, the duodenum, and pancreatic tissue, in order to avoid collateral damage and reduce intraoperative bleeding. The author believes that it is more convenient to use the electric coagulation hook when dissociating the cyst wall, as it not only provides a good operation angle, but also makes it possible to clearly separate stripped layers.

In laparoscopic total choledochal cyst resection, bleeding mostly occurs during stripping of the cyst. First, the wound surface of the cyst wall oozes blood, which is more common when there is severe adhesion between the cyst and surrounding tissue or severe inflammation of the cyst wall with obvious congestion and edema. If the hemostatic effect of the electric coagulation hook is not good, the tissue can be electrocoagulated by separation pliers to increase the area of electric coagulation. There is also potential for vascular injury around the cyst, for example, in the gallbladder artery, right hepatic artery, superior pancreaticoduodenal artery, and portal vein. Therefore, the intraoperative procedure should be performed gently, with careful differentiation of the anatomical structures based on clear visualization of the surgical field. In particular, when dissociating the proximal cyst, the distal cyst should be completely resected and the anterior wall of the proximal cyst should be suspended by suture traction. Further, the posterior wall of the proximal cyst should be carefully dissociated from the bottom to the top under direct observation to avoid portal vein injury.

End-to-end jejunal anastomosis is performed outside the body. Before anastomosis, an appropriate length of jejunal loop should be reserved; the length is determined based on the age and height of the child. At present, the straight-line distance between the umbilical nest and the hepatic porta is typically considered an appropriate length for the jejunal loop (15–25 cm) (18). During jejunal anastomosis, the author uses a 5–0 absorbable line to continuously suture the anterior and posterior walls of the intestinal tube to speed up anastomosis. No postoperative intestinal fistula occurred in any of the patients in this cohort. After the completion of anastomosis, the mesenteric hiatus should be closed. Further, it should be ensured that the proximal jejunum, distal jejunum, and jejunal loop are in the appropriate positions in relation to each other, and they should be integrated into the abdominal cavity in an orderly manner so as to avoid postoperative complications such as mesenteric hiatus hernia and mechanical intestinal obstruction.

Hepaticojejunostomy is the core step of the entire procedure. Continuous suture is performed with a 4–0 barbed suture. First, the posterior wall of the intestinal tube and common hepatic duct are sutured at 3 o’clock. The needle is passed through the coil at the end of the wire and is tightened to complete the first knot. Then, the posterior wall of the intestinal tube and the common hepatic duct are sutured continuously from left to right until the needle emerges at 9 o’clock. The anterior wall is then continuously sutured with a second barbed suture in the same way. Since the barbed suture can produce lasting and uniform tension when suturing tissue, it can be closely interlocked with the tissue after it passes through the tissue to avoid reverse slippage. Therefore, continuous suture does not require repeated tightening of the suture as it passes back and forth; this shortens the anastomosis time and ensures the tightness of the anastomosis (19). Good blood supply to the anastomosis is an important factor to avoid postoperative biliary fistula. The author believes that the use of the electric coagulation hook should be minimized when dissociating the proximal cyst and trimming the opening of the common hepatic duct, so as to reduce the influence of heat on blood supply to the bile duct wall. Especially when trimming the opening of the common hepatic duct, it is advisable to use scissors to cut and trim the edges neatly. In the process of trimming and anastomosis, repeated clamping and pulling of the bile duct wall should be minimized to protect blood supply to the bile duct wall to the greatest extent possible.

In this group, there were 2 cases of postoperative biliary fistula, which occurred in the early stage of the operation. Our team considered that the suture needle distance was too large during the biliary intestinal anastomosis, and no biliary fistula occurred after the needle distance was reduced in the later stage of the operation.

## Conclusion

Laparoscopic surgery for congenital choledochal cyst in children is safe and effective, and has the advantages of being minimally invasive, causing a low degree of trauma and bleeding, and ensuring quick postoperative recovery and an aesthetic appearance. It is important for the surgeon to be skilled in laparoscopic procedures and to pay attention to the intraoperative handling of the various instruments to ensure safe and effective completion of the surgery and reduce postoperative complications.

## Data Availability

The authors acknowledge that the data presented in this study must be deposited and made publicly available in an acceptable repository, prior to publication. Frontiers cannot accept a manuscript that does not adhere to our open data policies.
